# Analysis of the multiple drivers of vegetation cover evolution in the Taihangshan-Yanshan region

**DOI:** 10.1038/s41598-024-66053-6

**Published:** 2024-07-03

**Authors:** Feng Yan, Xinyu Guo, Yuwen Zhang, Jing Shan, Zihan Miao, Chenyang Li, Xuehan Huang, Jiao Pang, Yaheng Chen

**Affiliations:** 1 School of Water Resources and Environment, China University of Geosciences (Beijing), Beijing, 100083 China; 2 School of Land and Resources, Hebei Agricultural University, Baoding, 071001 China; 3 College of Natural Resources and Environment, Northwest A&F University, Yangling, 712100 China; 4 School of Modern Science and Technology, Hebei Agricultural University, Baoding, 071001 China; 5 Bohai College, Hebei Agricultural University, Huanghua, 061100 China

**Keywords:** FVC, Taihangshan-Yanshan, Spatio-temporal succession, Drivers, Human activities, Climate-change ecology, Environmental sciences

## Abstract

The Taihangshan-Yanshan region (TYR) is an important ecological barrier area for Beijing-Tianjin-Hebei, and the effectiveness of its ecological restoration and protection is of great significance to the ecological security pattern of North China. Based on the FVC data from 2000 to 2021, residual analysis, parametric optimal geodetector technique (OPGD) and multi-scale geographically weighted regression analysis (MGWR) were used to clarify the the multivariate driving mechanism of the evolution of FVC in the TYR. Results show that: (1) FVC changes in the TYR show a slowly fluctuating upward trend, with an average growth rate of 0.02/10a, and a spatial pattern of "high in the northwest and low in the southeast"; more than half of the FVC increased during the 22-year period. (2) The results of residual analysis showed that the effects of temperature and precipitation on FVC were very limited, and a considerable proportion (80.80% and 76.78%) of the improved and degraded areas were influenced by other factors. (3) The results of OPGD showed that the main influencing factors of the spatial differentiation of FVC included evapotranspiration, surface temperature, land use type, nighttime light intensity, soil type, and vegetation type (q > 0.2); The explanatory rates of the two-factor interactions were greater than those of the single factor, which showed either nonlinear enhancement or bifactorial enhancement, among which, the interaction of evapotranspiration with mean air and surface temperature has the strongest effect on the spatial and temporal evolution of FVC (q = 0.75). Surface temperature between 4.98 and 10.4 °C, evapotranspiration between 638 and 762 mm/a, and nighttime light between 1.96 and 7.78 lm/m^2^ favoured an increase in vegetation cover, and vegetation developed on lysimetric soils was more inclined to be of high cover. (4) The correlation between each variable and FVC showed different performance, GDP, elevation, slope and FVC showed significant positive correlation in most regions, while population size, urban population proportion, GDP proportion of primary and secondary industries, and nighttime light intensity all showed negative correlation with FVC to different degrees. The results can provide data for formulating regional environmental protection and restoration policies.

## Introduction

Vegetation is an important component of terrestrial ecosystems and is a key hub connecting the atmosphere, hydrosphere, and the soil circle in terms of material cycling and energy flow^1^. Vegetation cover (FVC) refers to the percentage of the vertical projection area of above-ground vegetation organs on the ground to the total area of vegetation^2^, which can reflect the growth condition of regional surface vegetation and the quality of ecological environment. In several studies, global vegetation cover has shown an increasingly significant increasing trend^3^, especially in the middle and high latitudes of the Northern Hemisphere^4^ Studies conducted in the Chinese region have reached the same conclusion^5^. The overall FVC of grassland in Inner Mongolia shows the distribution of east-high and west-low as well as the fluctuating and rising development trend, and the grassland in Inner Mongolia will develop positively and sustainably in the future^6^. Vegetation cover in the Yellow River Basin showed a significant growth trend, and environmental variables such as temperature, precipitation and CO_2_ concentration positively promoted vegetation growth^7^. The vegetation growth process is significantly constrained by natural conditions, and among the meteorological factors, temperature and precipitation are considered to be the main factors affecting vegetation cover^8^, and excessive human activities can interfere with vegetation suitability^9^, especially in the context of rapid urbanisation, the continuous expansion of urban land will inevitably result in the transformation of land-use patterns, which inevitably take up of arable land and transform it into construction land, resulting in vegetation degradation. It is worth noting that under the active implementation of ecological protection projects, the effect of vegetation restoration is remarkable^10^, further reflecting the two-sided nature of vegetation response to human activities. However, the impacts of climate change and human activities on FVC have not been fully quantified^11^, and it is of great significance to strengthen the monitoring of vegetation cover changes in the region and explore the driving mechanisms of vegetation changes. In addition, NDVI is also a parameter applied to characterise vegetation cover, and its trend can respond to changes in vegetation cover, productivity and health^12^, but the NDVI equation itself has the potential flaw of being easily saturated, and is limited in its ability to deal with atmospheric disturbances, and, in addition, it is also affected by background disturbances in the soil and leaf canopy^13^. Numerous studies have shown that there is a significant linear correlation between NDVI and FVC^14^. Therefore, FVC data were chosen to start the analysis in this paper.

Residual analysis method, as a commonly used quantitative analysis tool, has been widely used in the study of vegetation change driver analysis.The change of NPP in Shaanxi Province is subject to two drivers, climate and human activities, while the main driver is human activities^15^, which not only promotes the vegetation cover, but also has a destructive effect on the vegetation^16^. The residual analysis method can accurately reveal the effects of climatic conditions and other factors in regional vegetation dynamics^17^, but most of the other factors are attributed to the role of human activities, ignoring the effects of topographic factors, soils, and other factors on vegetation, and this linear traditional model cannot quantify significant drivers and their explanatory ability for FVC, and again tends to ignore synergistic or antagonistic effects. To solve this problem, many scholars have carried out useful explorations. Geodetector (GD)^18^ is a set of statistical methods for detecting spatial dissimilarity and revealing the driving forces behind it^19^, which contains four modules, namely, factor detection, risk detection, interaction detection, and ecological detection^20^, but traditional geodetectors need to manually discretise continuous data, and the q-value will be different depending on the method, number, and sampling scale of the discretisation. However, the traditional geoprobes need to discretise the continuous data manually, and the q-value will be different depending on the discretisation method, number and sampling scale. The optimal parameter geoprobe model (OPGD) solves these shortcomings and improves the accuracy of spatial analysis by automatically discretising the parameters optimally through a procedure^21^. This model is now widely used, such as studying the effects of each driver on vegetation and the interaction between them^22,23^, exploring the drivers of flash floods in Jiangxi Province^24^, and carrying out the research on the drivers of the source of heavy metals in arable soils and their interaction^25^. In the process, we also took into account the spatially non-stationary characteristics of the independent and dependent variables and applied the multi-scale geographically weighted regression (MGWR) spatial statistical model, thus revealing the complex local relationships from a spatial perspective^26^. Multiscale geographically weighted regression (MGWR) is an improvement of geographically weighted regression model (GWR), which generates multiple adaptive bandwidths at different spatial scales with a good level of spatial smoothing^27^. In recent years, the MGWR model has been successfully applied to the analysis of impact mechanisms such as urban traffic^28^ and heat island effect^29^, and partial correlation analysis is usually used in previous studies to elaborate the correlation between FVC and meteorological factors^30–33^, but the partial correlation calculation will become exceptionally cumbersome when there are more variables, while MGWR can detect the correlation between multiple driving factors and FVC, and can elaborate its spatial heterogeneity from a multi-scale perspective.

Ecological projects implemented in China are recognised as the most effective afforestation and conservation projects^34^. As an important mountain range in North China, Taihangshan-Yanshan Mountain is an important ecological barrier area and water conservation area in Beijing-Tianjin-Hebei, its ecological environment is very fragile, and research on the spatial and temporal characteristics of its ecosystems and dynamic changes is relatively weak, and despite the fact that local relevant departments have paid great attention to ecological restoration projects, a good balance between economic development and green ecological protection has not yet been achieved However, few scholars have studied the vegetation cover in the TYR, and there is a lack of analysis of the multi-dimensional driving mechanism of vegetation change. To fill this gap, this paper uses one-dimensional linear fitting and Sen trend analysis based on the long time-series of FVC data from 2000 to 2021 to assess the temporal and spatial characteristics and dynamics of the FVC in the TYR, which is a relatively weak study. In order to fill the gap, this paper analyses the spatial and temporal characteristics of FVC in the TYR over the past 22 years based on the long time series of FVC data from 2000 to 2021, using univariate linear fitting and Sen trend analysis to characterize the spatial and temporal changes of FVC in the past 22 years, and uses the residual analysis to quantify the relative contributions of meteorological and other factors to the evolution of FVC, and further identifies and explores the multivariate drivers and their spatial heterogeneity by combining with the OPGD and the MGWR, and the research results can provide important The results of this study can provide an important reference for monitoring vegetation change, ecological environmental protection and restoration in the TYR.

## Research data and methodology

### Overview of the study area

**Figure 1:**
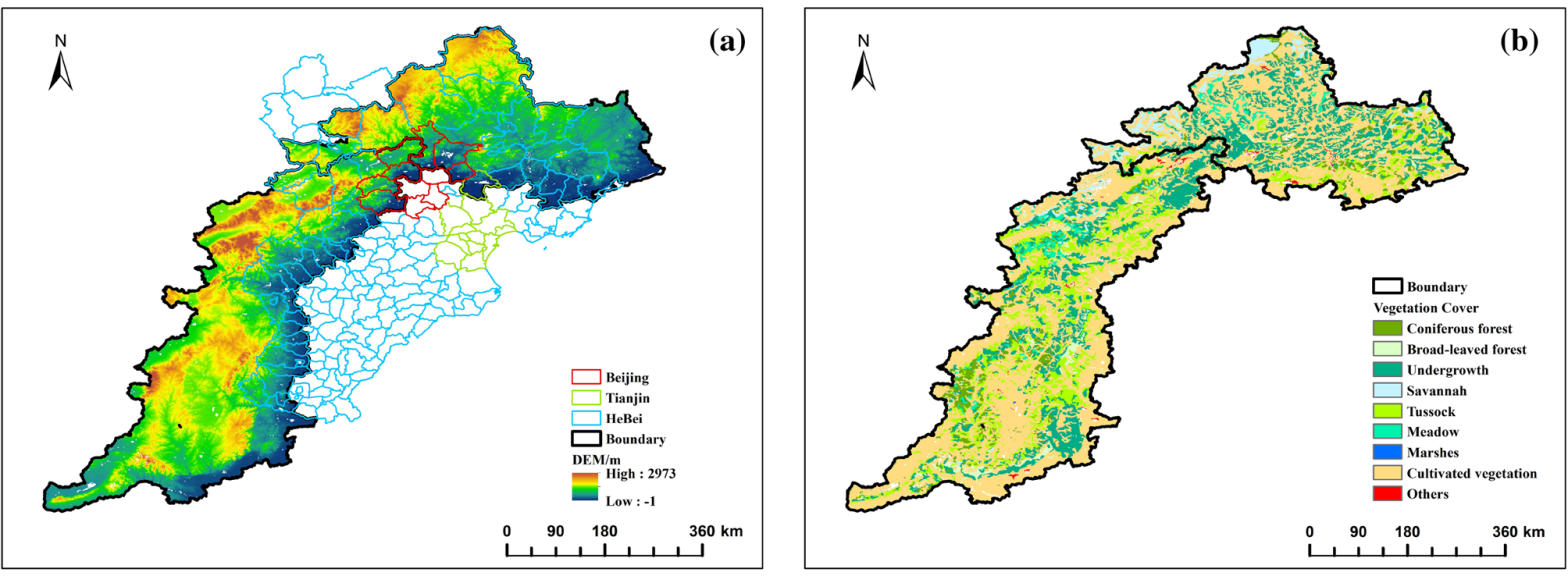
DEM map and vegetation type map of the research area.

The Taihang Mountains (34°34ʹ–41°06ʹN, 110°13ʹ–114°33ʹE) cover a total area of about 127,000 km^2^, with a general south-west-north-east orientation. It spans four provinces and cities, Henan, Shanxi, Hebei and Beijing, from south to north, and stretches for more than 400 kms. The terrain is high in the north and low in the south (as shown in Fig. 1), with mainly deciduous broad-leaved forests and thickets, among which there are sparse oil pine forests. The climate is continental, with an average annual temperature of 10.3 °C and an annual precipitation of 501.8 mm. The saline and alkaline land in the area is large, and drought, flooding and alkalinity co-exist in the arable land, with tamarisk, reed and verbena as the main plants; the Yanshan Mountain Range (39°40ʹ–42°10ʹN, 115°45ʹ–119°50ʹE) is located in the northern part of Hebei Province, and is roughly east–west orientated. The Yanshan Mountains are part of the Yanshan Sinking Belt, and are the birthplace and confluence of many rivers, including the Luan River and the Chaobai River. It is in the warm temperate continental monsoon climate zone, with an average annual temperature of 6–10 °C and an annual precipitation of about 700 mm.

### Data acquisition and pre-processing

#### Vegetation coverage

The data used in the study is MODIS NDVI dataset on GEE platform, which has a temporal resolution of 10 days and a spatial resolution of 500 m. The time range of the selected images is from 2000 to 2021. The image processing was mainly realised by script programming through the GEE cloud platform, applying the maximum synthetic value method and the image element dichotomous model to transform the NDVI, and finally obtaining the FVC (vegetation cover) data with the resolution changed to 1 km. The maximum synthetic value method was used to calculate the annual and quarterly FVC values respectively.

The formula for the like element dichotomy is:

1 $${\text{FVC }} = \, \left( {{\text{S }} - {\text{ S}}_{{{\text{soil}}}} } \right) \, \left( {{\text{S}}_{{{\text{veg}}}} - {\text{ S}}_{{{\text{soil}}}} } \right)$$

where S is a single image element; S_veg_ is vegetation information; S_soil_ is soil information; and FVC is vegetation cover.

In this study, NDVI was set as individual image information, NDVI_soil_ was set as the image cumulative approximately equal to 5% of the image's NDVI value, i.e. soil information, and NDVI_veg_ was set as the image cumulative 95% of the image's NDVI value, i.e. vegetation information^35^. Equation (1) can be expressed as

2 $${\text{FVC }} = \, \left( {{\text{NDVI }} - {\text{ NDVI}}_{{{\text{soil}}}} } \right) \, \left( {{\text{NDVI}}_{{{\text{veg}}}} - {\text{ NDVI}}_{{{\text{soil}}}} } \right)$$

#### Meteorological data

The meteorological data come from the National Earth System Science Data Centre (http://www.geodata.cn) and are generated by downscaling in the Chinese region through the Delta spatial downscaling programme, based on the global 0.5° climate data released by CRU and the global high-resolution climate data released by WorldClim. The time span is 1901–2021, and the spatial resolution is 0.0083333° (about 1 km). The unit of temperature is 0.1 °C, and the unit of precipitation is 0.1 mm. The data, which were downloaded in nc format, were imported into ArcGIS, and further processed to obtain the monthly average temperature/precipitation and annual average temperature/precipitation data by creating NetCDF raster layers, creating raster layers, and etc. The data were then processed to obtain the monthly average temperature/precipitation and annual average temperature/precipitation data. The raster image dataset with the same pixel size and the same coordinate system as the FVC data was obtained by unifying Taihangshan the coordinate system to WGS 1984 with the Definitional Projection Tool after mask cropping according to the vector boundary of -Yanshan region.

### Methods of data analysis

#### Sen trend analyses

As a non-parametric test, the Mann–Kendall test is a widely used test for trend. It does not require the data to follow a certain distribution and is not affected by extreme values and outliers. For the FVC time series with a sample size of n = 22, the Z statistic is transformed from the S statistic to test the significance of the trend, as shown in Eqs. (3)–(7).

3 $${\text{FVC}}_{{\text{i}}} .{\text{i }} = { 2}000,{ 2}00{1}, \, . \, . \, . \, ,{ 2}0{21}$$

4 $$\text{Z }=\left\{\begin{array}{ll}\frac{\text{S}-1}{\sqrt{\text{Var}(\text{S})}}, & \quad S>0\\ 0, & \quad S=0\\ \frac{\text{S}+1}{\sqrt{\text{Var}(\text{S})}},& \quad S<0\end{array}\right.$$

5 $$\text{S}=\sum_{\text{j}=1}^{\text{n}-1}\sum_{\text{i}=\text{j}+1}^{\text{n}}\text{sgn }({\text{FVC}}_{\text{j}} - {\text{FVC}}_{\text{i}})$$

6 $$\text{sgn}({\text{FVC}}_{\text{j}}-{\text{FVC}}_{\text{i}})=\left\{\begin{array}{ll}1, & \quad {\text{FVC}}_{\text{j}}-{\text{FVC}}_{\text{i}}>0\\ 0, & \quad {\text{FVC}}_{\text{j}}-{\text{FVC}}_{\text{i}}=0\\ -1, & \quad {\text{FVC}}_{\text{J}}-{\text{FVC}}_{\text{i}}<0\end{array}\right.$$

7 $$Var(S)=\frac{n(n-1)(2n+5)}{18}$$

where Var is the variance; sgn is the sign function; FVC_j_ and FVC_i_ denote the FVC values in year j and year i; and Z and S are the test statistics.Mann and Kendall proved that the statistic S roughly obeys the normal distribution when n ≥ 8, and Z is the standard normal distribution test statistic for S. Theil-Sen median trend analysis was used to detect the trend of vegetation cover change and to test for its significance. In this study, the Theil-Sen median trend analysis and the Mann–Kendall test were combined to detect trends in vegetation cover and test for significance.

#### Residual analysis

Residual analyses allow quantitative separation of the relative contribution of climatic and other factors (mainly human activities, topography, natural hazards, etc.) to changes in vegetation cover^36^. In this paper, a binary primary linear regression model between the actual FVC and the temperature and precipitation elements is developed in Matlab software as a way to find out the portion of the FVC that is affected by climatic factors, i.e., the predicted FVC. The difference between the predicted FVC and the actual FVC is used to measure the magnitude of increase or decrease in FVC due to other activities, calculated as shown in equation:

8 $${\text{FVC}}_{{\text{O}}} = {\text{FVC}}_{{\text{A}}} - {\text{FVC}}_{{\text{P}}}$$

where FVC_A_ is the actual FVC; FVC_P_ is the predicted FVC; and FVC_O_ is the residual, i.e., the value of the loss or gain in FVC influenced by factors other than precipitation and temperature. If FVC_O_ is positive or negative, it represents that other factors have a positive or negative effect on FVC changes.

Regression analysis was performed between FVC and temperature and precipitation with the regression equation:

9 $${\text{FVC}}_{{\text{p}}} = {\text{a}}*{\text{pre}} + {\text{b}}*{\text{tem}} + {\text{c}}$$

The Mann–Kendall test is applied to analyse FVC_O_, FVC_A_ and FVC_P_ to better study the trend of the impact of other activities on FVC, and to derive their changes in FVC from 2000 to 2021, respectively: K_O_, K_A_, K_P_, i.e., the three slopes of FVC under the effect of other factors, actual FVC and FVC under climate change, and if K < 0, it indicates that FVC shows a decreasing trend in the study period, and vice versa. If K < 0, it indicates that FVC shows a decreasing trend during the study period, and vice versa, it shows an increasing trend. the absolute value of K reflects the degree of change, the larger the absolute value, the stronger the change, and vice versa, the smaller the change. The following scenarios (Table 1) are formed by combining the slopes of the three FVCs above to determine the effects of climate change and other factors on FVC changes in the TYR.

**Table 1:** Assess the relative roles of climate change and other factors in changing FVC under different scenarios.

Change of type	Scenario type	K_P_	K_O_	Relative role of climate change/%	Relative role of other factors/%	Instruction
Vegetation improvement area (K_A_ > 0)	Scenario 1	> 0	> 0	$\frac{\left|{K}_{P}\right|}{\left|{K}_{P}\right|+\left|{K}_{O}\right|}\times 100$	$\frac{\left|{K}_{O}\right|}{\left|{K}_{P}\right|+\left|{K}_{O}\right|}\times 100$	All factors combine to promote improved vegetation cover
	Scenario 2	> 0	< 0	100	0	Climate change alone promotes improved vegetation cover
	Scenario 3	< 0	> 0	0	100	Other factors individually contributing to improved vegetation
Vegetation degradation area (K_A_ < 0)	Scenario 4	< 0	< 0	$\frac{\left|{K}_{P}\right|}{\left|{K}_{P}\right|+\left|{K}_{O}\right|}\times 100$	$\frac{\left|{K}_{O}\right|}{\left|{K}_{P}\right|+\left|{K}_{O}\right|}\times 100$	Combination of all factors contributing to vegetation degradation
	Scenario 5	< 0	> 0	100	0	Climate change alone contributes to vegetation degradation
	Scenario 6	> 0	< 0	0	100	Other factors individually contributing to vegetation degradation

#### Parameter-optimised geodetector technology

1. Parameter optimisation
  Using an R language program, the q-value of each driver was calculated for different classification methods (equal interval classification, natural breakpoints, quartiles, geometric spacing, and standard deviation classification) and number of classifications (3 to 9 classes), and the combinations with the highest q-value of the drivers were screened for optimal spatial discretisation^37^.
2. Factor detection
  The factor detection formula is as follows:
  10 $${\text{q}} = 1 - \frac{{\sum\nolimits_{{\text{h}} = 1}^{\text{L}} {{{\text{N}}_{\text{h}}}\upsigma _{\text{h}}^2} }}{{{\text{N}}{\upsigma ^2}}} = 1 - \frac{{{\text{SSW}}}}{{{\text{SST}}}}$$
  where q is a measure of the explanatory power of the independent variable, q is between 0 and 1, the larger q is, the stronger the explanatory power; L is the number of strata of the influencing factors; ${\text{N}}_{\text{h}}$ is the number of units in the layer;N is the number of units in the region; ${\upsigma }_{\text{h}}^{2}$ is the variance of the change in FVC in layer h; ${\upsigma }^{2}$ is the variance of FVC change across the region. SSW is the sum of within-stratum variance; SST is the total variance across the region.
3. Interaction detection
  The q-values of two-factor interactions can be categorised into five types (Table 2).

**Table 2:** Types of interactions between two variables.

Interaction	Basis of judgement
Nonlinear attenuation	$\text{q}(\text{X}1\cap \text{X}2)<\text{Min}(\text{q}\left(\text{X}1\right),\text{q}\left(\text{X}2\right))$
Single-factor nonlinear attenuation	$\text{Min}(\text{q}\left(\text{X}1\right),\text{q}\left(\text{X}2\right))<\text{q}(\text{X}1\cap \text{X}2)<\text{Max}(\text{q}\left(\text{X}1\right),\text{q}\left(\text{X}2\right))$
Two-factor enhancement	$\text{q}(\text{X}1\cap \text{X}2)>\text{Max}(\text{q}\left(\text{X}1\right),\text{q}\left(\text{X}2\right))$
Independent	$\text{q}(\text{X}1\cap \text{X}2)=\text{q}(\text{X}1)+\text{q}(\text{X}2)$
Nonlinear enhancement	$\text{q}(\text{X}1\cap \text{X}2)>\text{q}(\text{X}1)+\text{q}(\text{X}2)$
4. Ecological detection
  Ecological detection is used to compare whether the independent variables X1 and X2 have a significant effect on the spatial distribution of the dependent variable Y, as measured by the F statistic
  11 $$\text{F}=\frac{{\text{N}}_{\text{X}1}({\text{N}}_{x2}-1){\text{SSW}}_{\text{X}1}}{{\text{N}}_{\text{X}2}({\text{N}}_{x1}-1){\text{SSW}}_{\text{X}2}}$$
  12 $${SSW}_{X1}=\sum_{h=1}^{L1}{N}_{h}{\sigma }_{h}^{2},\;\;\;{SSW}_{X2}=\sum_{h=1}^{L2}{N}_{h}{\sigma }_{h}^{2}$$
  where ${\text{N}}_{\text{X}1}\text{ and }{\text{N}}_{\text{X}2}$ denote the sample sizes for factors X1 and X2 respectively; ${\text{SSW}}_{\text{X}1}\text{ and }{\text{SSW}}_{\text{X}2}$ denote the sum of the intra-layer variances of the strata formed by and , respectively;$\text{L}1\text{ and L}2$ Number of variables and strata, respectively .where the null hypothesis ${\text{H}}_{0}$: ${\text{SSW}}_{\text{X}1}={\text{SSW}}_{\text{X}2}$
5. Risk detection
  Risk area detection is used to assess whether there is a significant difference in the average characteristics between sub-areas of a factor, a method that facilitates the identification of areas with good vegetation cover. During risk area detection, the following tests were performed using the t-statistic:
  13 $${t}_{\overline{y }h=1-\overline{y }h=2}=\frac{{\overline{Y} }_{h=1}-{\overline{Y} }_{h=2}}{{\left[\frac{Var\left({\overline{Y} }_{h=1}\right)}{{n}_{h=1}}+\frac{Var\left({\overline{Y} }_{h=2}\right)}{{n}_{h=2}}\right]}^{1/2}}$$
  ${\overline{\text{Y}} }_{\text{h}}$ indicates the mean value of the attribute in the subregion; ${\text{n}}_{\text{h}}$ is the number of samples in subregion h;Var denotes variance.

#### Multi-scale geographically weighted regression analysis MGWR

The multi-scale geographically weighted regression (MGWR) model can effectively articulate spatial correlation and non-stationarity, and is calibrated using a backward fitting algorithm, which further takes into account the different bandwidths of the independent variables, unlike the traditional geographically weighted regression (GWR) model, and makes up for the lack of optimal bandwidth limitation by dividing the independent variables at global and local scales, flexibly identifying the scaling problem of the influence of the different variables, and guaranteeing the reliability of the overall model, reflecting spatial heterogeneity in the influence factors and FVCs^38^ The formula is as follows:

14 $${\text{y}}_{\text{i}={\upbeta }_{0}}({\text{u}}_{\text{i}},{\text{v}}_{\text{i}})+\sum_{\text{j}=1}^{\text{k}}{\upbeta }_{\text{bwj}}({\text{u}}_{\text{i}},{\text{v}}_{\text{i}}){\text{x}}_{\text{ij}}+{\upvarepsilon }_{\text{i}}$$

In the formula, ${y}_{i}$ denotes the FVC fitted value of the sample.${x}_{ij}$ is the value of the $\text{j}$ independent variable in sample $\text{i}{\beta }_{0}({u}_{i},{v}_{i})$ is a constant term for the geographic coordinates in which each variable is located.${\beta }_{bwj}({u}_{i},{v}_{i})$ representing the spatial location of the first variable fitted using a particular regression bandwidth.$\text{j}$ th spatial location of the first variable fitted using a particular regression bandwidth. ${\varepsilon }_{i}$ is obeying a normal distribution error with zero mean.

## Result and analysis

### Temporal and spatial patterns of vegetation coverage in the study area

#### Analysis of FVC spatiotemporal scale change

**Figure 2:**
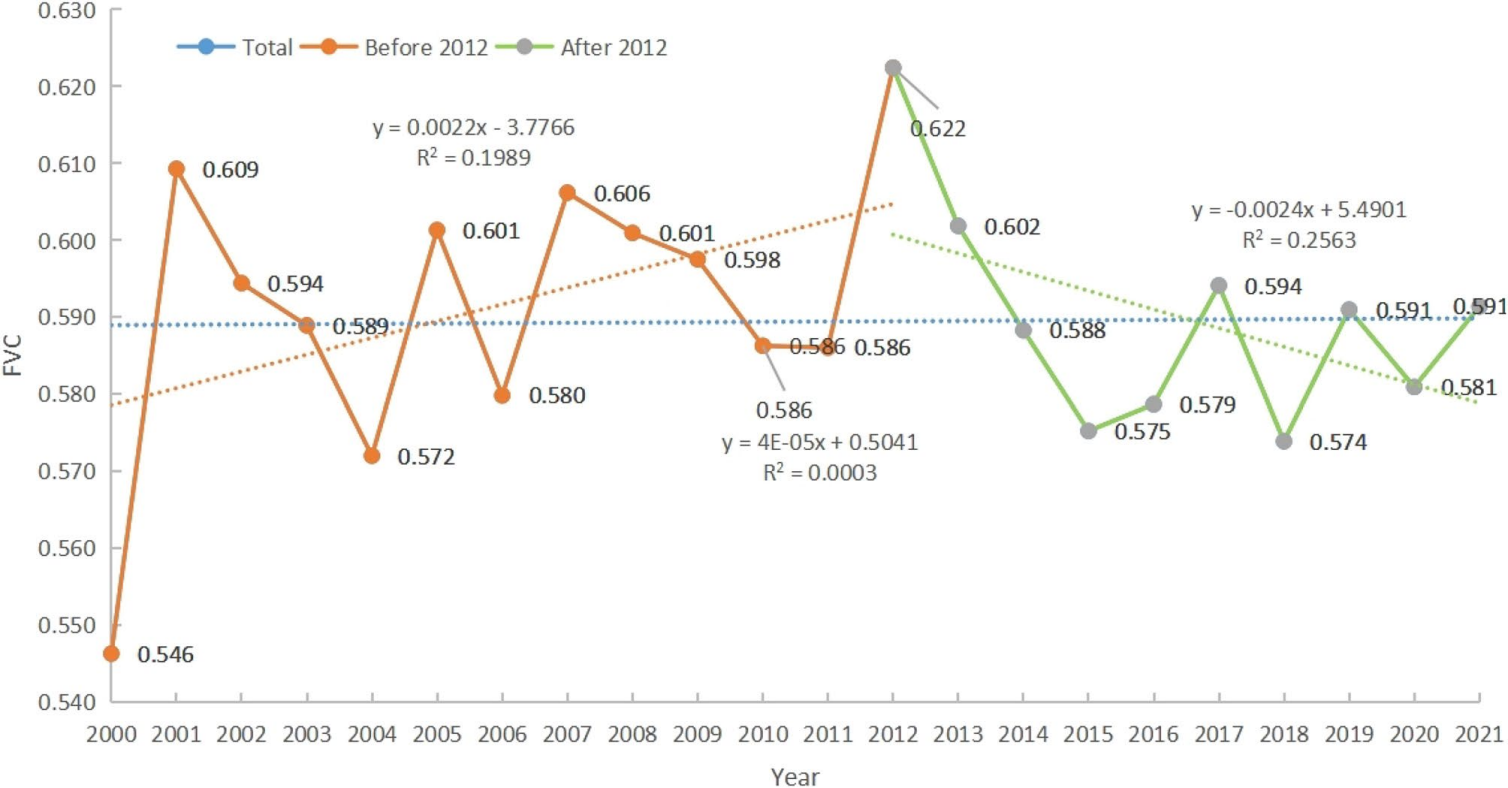
Temporal variation map of vegetation's annual FVC from 2000 to 2021.

Figure 2 shows that the average annual FVC value for 2000–2021 ranged from 0.540 to 0.630, with an average FVC value of 0.589 for 22a. Before 2012, there was a linear rate of increase of 0.0022/a, while after 2012, there was a linear decline rate of 0.0024/a. The overall performance displays a weak upward trend. The FVC value in 2000 was the lowest at 0.546, which may be attributed to the region's industrial development and disregard for the ecological environment after the reform and opening up. The highest FVC value was recorded in 2012 at 0.622, which may be attributed to the Chinese government's emphasis on ecological civilization construction.

**Figure 3:**
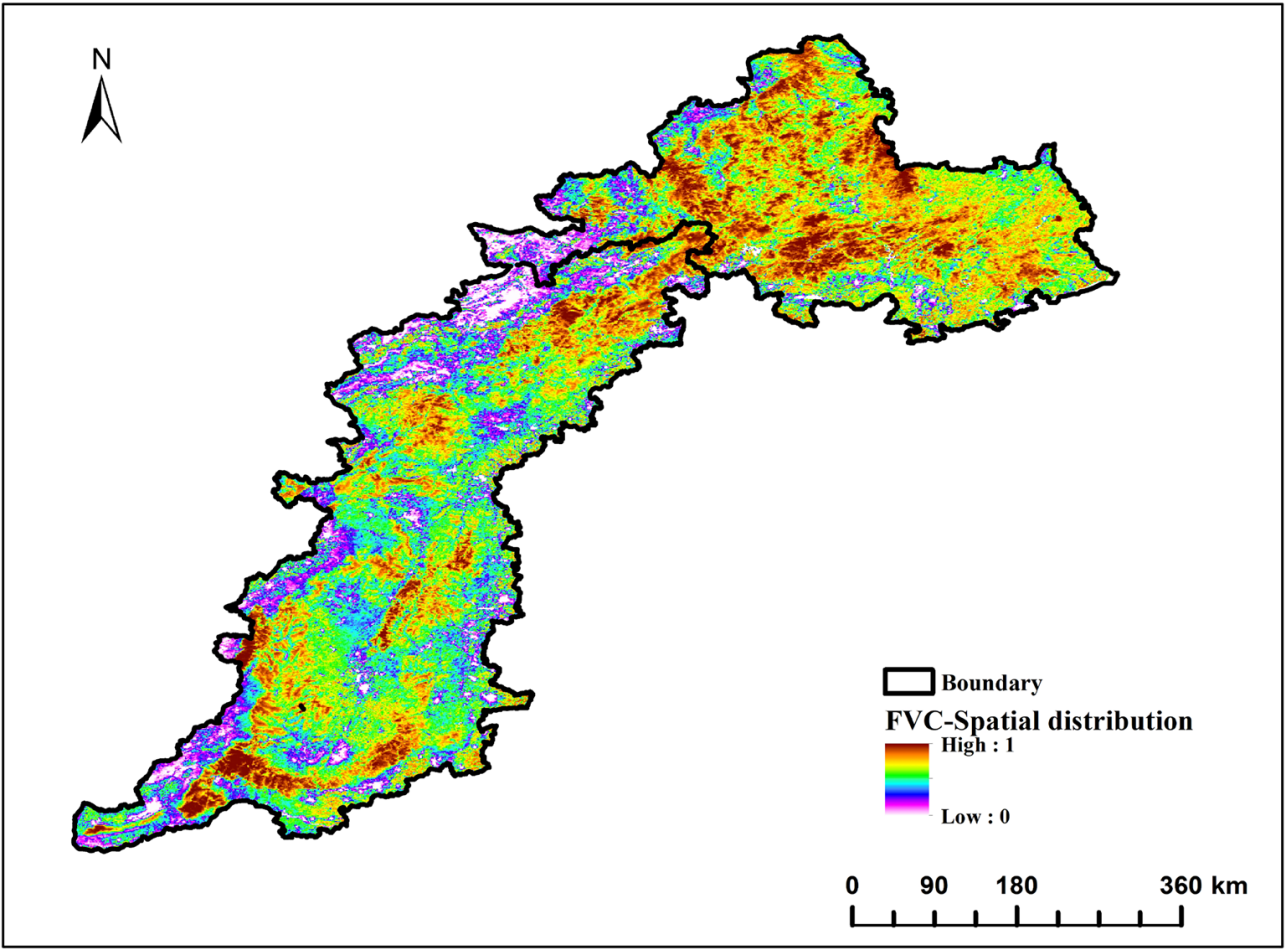
Spatial distribution of the annual mean of the FVC.

The annual mean FVC values follow a pattern of being high in the north and south, and low in the middle, as shown in Fig. 3. There is a clear decrease in FVC in the area where Taihang Mountain and Yanshan Mountain meet, as well as in the western region of Taihang Mountain. The regions with high average values are Chengde City, Jincheng City, and some areas of Beijing and Baoding City. This may be attributed to the long-term construction of ecological environmental protection and the development of green agriculture in the local area. The region with a low average value is located near the Loess Plateau and Inner Mongolia Plateau. It belongs to the temperate continental monsoon climate, where evaporation exceeds precipitation, resulting in a dry climate, low vegetation coverage, and a poor ecological environment. The FVC value in the southernmost part of Taihang Mountain is higher on average. This may be due to the dominance of evergreen forests such as Pinus tabulaeformis, platycypress, and white bark pine in the area.

#### Distribution of FVC trends pixel by pixel

**Figure 4:**
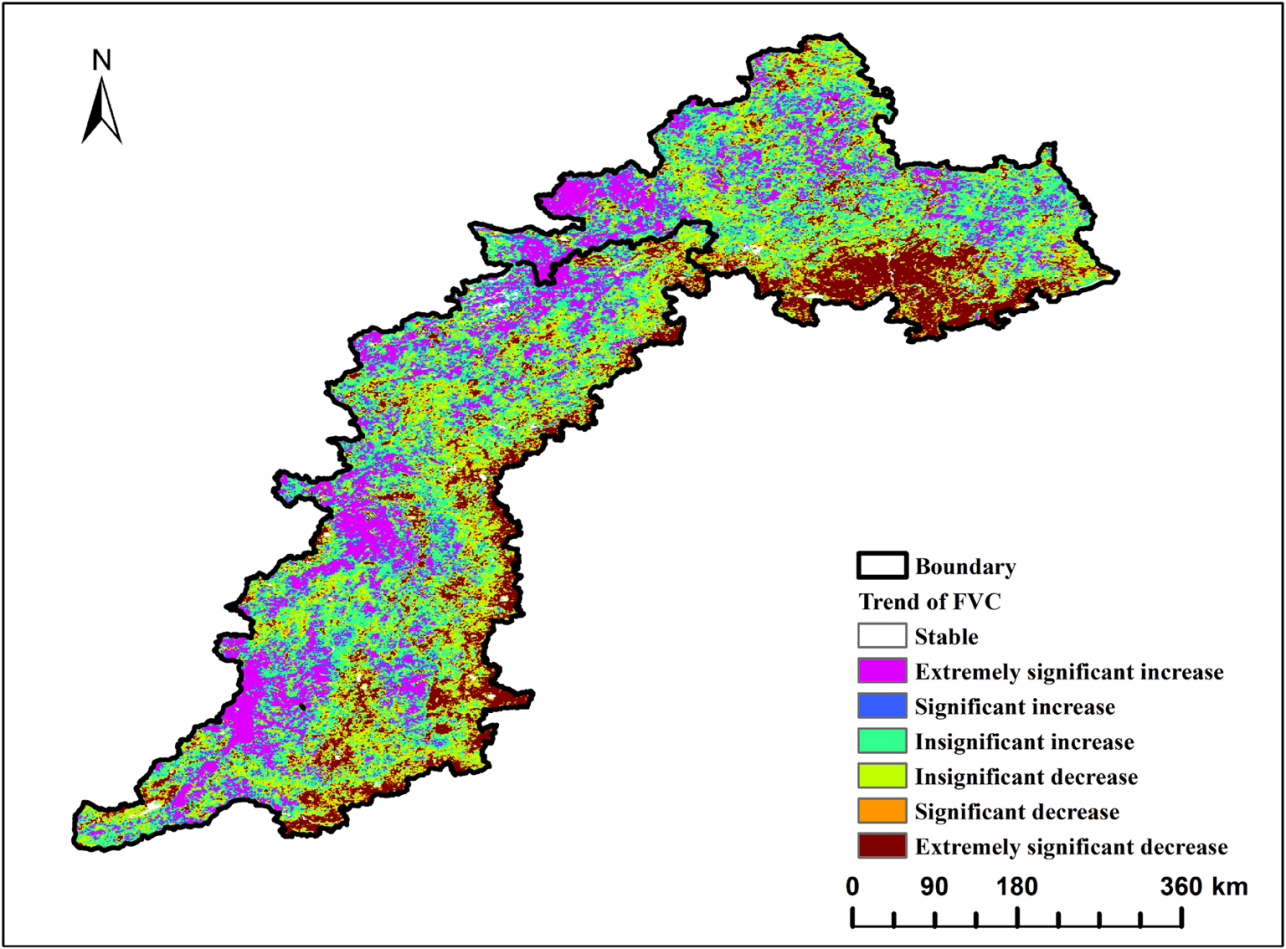
FVC trend significance zoning map.

This study categorises changes in FVC in 22a as: significant increase, very significant increase, no significant increase, basically stable, significant decrease, very significant decrease, and no significant decrease. Figure 4 shows that the change in FVC decreased gradually from west to east in the study area. The stable area accounted for 1.28% and was mainly distributed in Haigang District of Qinhuangdao City, Jizhou District of Tianjin City, and Miyun District of Beijing City. In Linfen City, Jinzhong City, and the Inner Mongolia Plateau bordering Yanshan and Taihang Mountains, FVC increased by 54.1%. Of these regions, 16.16% experienced an extremely significant increase, 8.46% experienced a significant increase, and 29.48% experienced an insignificant increase. The area of decrease accounted for 44.62%, with 23.6% in the eastern part of Taihang Mountain, 5.38% in the southern part of Yanshan Mountain, and 15.64% in Qinglong Manchu Autonomous County of Qinhuangdao. Overall, the area with an increasing trend of vegetation coverage in the TYR is larger than the area with a stable or decreasing trend.

### Analysis of the driving factors behind the spatiotemporal differentiation of FVC

#### The relative contribution of climate change and other factors to the evolution of FVC

**Figure 5:**
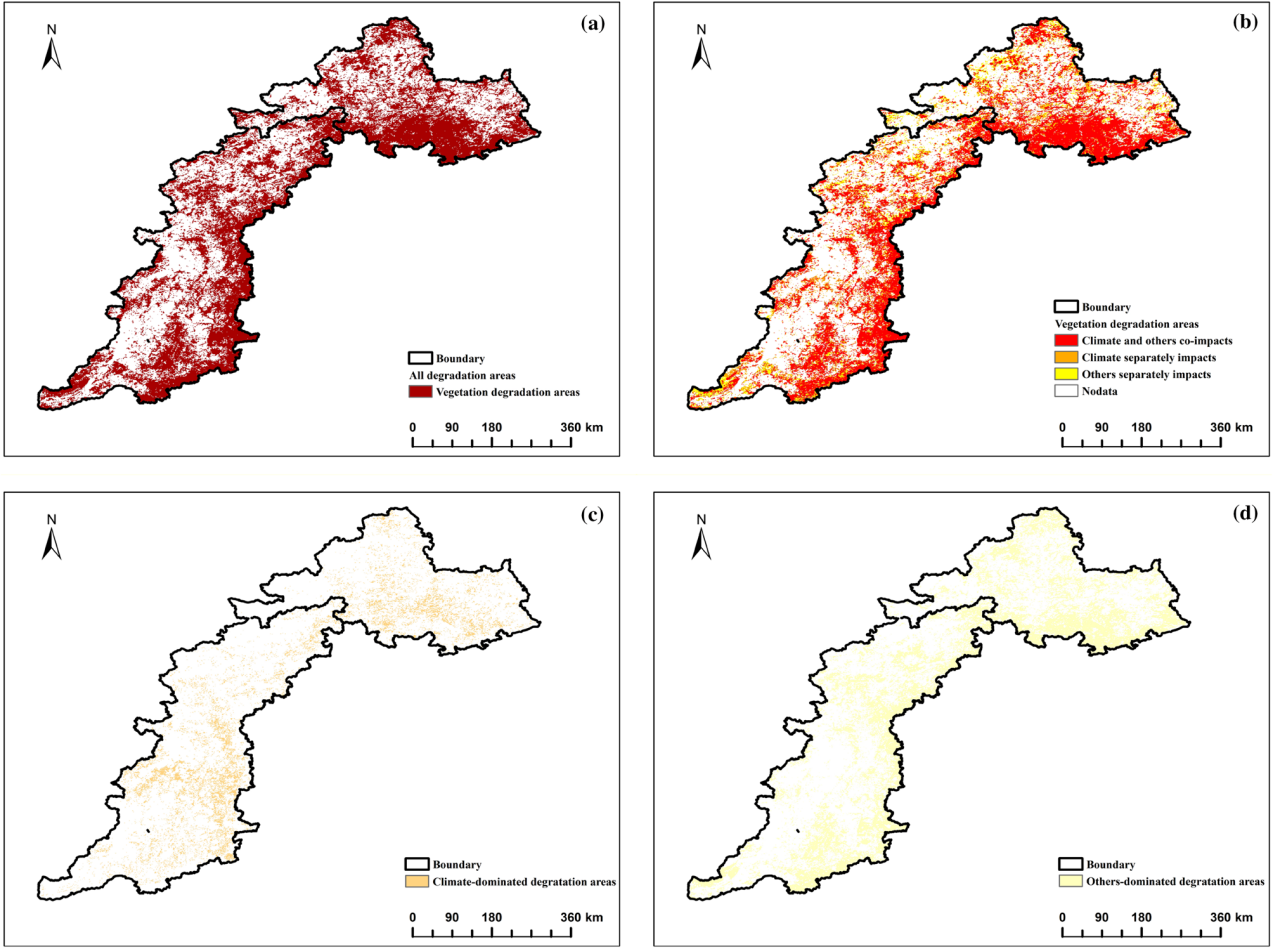
Relative impacts of climate change and other factors on FVC degraded areas.

**Figure 6:**
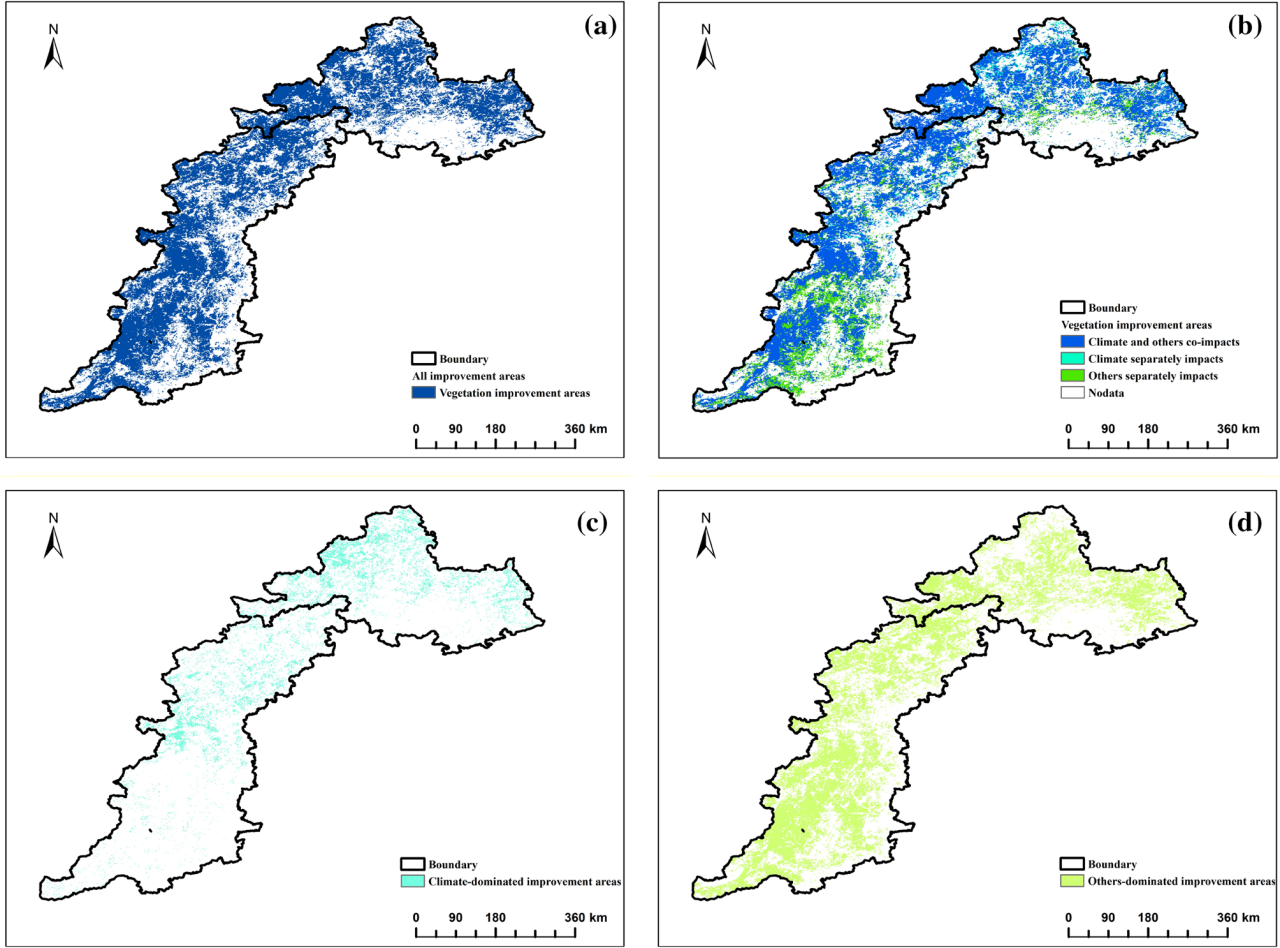
Relative impact of climate change and other factorson FVC improvement areas.

Based on the calculation method presented in Table 1, we obtained the distribution pattern of improved and degraded areas, as well as the relative effects of climate change and other factors on FVC change in the TYR. The vegetation degradation area accounted for 47.56%, while the improvement area accounted for 51.26%. Our analysis, combined with Figs. 5 and 6, indicates that the spatio-temporal differentiation pattern of FVC is less affected by climate factors and greatly influenced by non-climatic factors.

The area of FVC degradation is concentrated in the southern part of Yanshan and the eastern part of Taihang Mountains (Fig. 5a). Only 11.02% of this area is affected by climate change alone, which is approximately 23,981 km^2^ (Fig. 5b). It is mainly distributed in Xinglong County, Luanping County, Kuancheng Manchu Autonomous County, Laishui County, Jingxing County, Yushe County, Shahe City, Wuan City, Weihui City, and other locations. Only 23.22% of FVC changes were attributed to climate (Fig. 5c), while the remaining 76.78% were attributed to other factors (Fig. 5d). These factors were mainly distributed in Fengning Manchu Autonomous County, Weichang Manchu and Mongolian Autonomous County, Jianchang County, Shunping County, Huaian County, Xingtang County, Lingshou County, Yanhu District, Yongji City, Mengzhou City, and other locations.

FVC improvement areas are distributed throughout the north-central Yanshan and Taihang Mountains (Fig. 6a). Climate change alone accounts for only 6.15% of this distribution (Fig. 6b), covering an area of approximately 13,383 km^2^. These areas are mainly found in Fengning Manchu Autonomous County, Weichang Manchu Mongolian Autonomous County, Jianchang County, Suizhong County, Chicheng County, Xingtang County, Yi County, Fanshi County, Yu County, and other locations. The FVC changes were driven by climate in 19.20% of the area (Fig. 6c), while the remaining 80.80% was influenced by other factors (Fig. 6d). The climate-dominated area was mainly located in the northern Taihang Mountains and Yanshan Mountains. The areas dominated by other factors were mainly distributed in Luanping County, Miyun District, Lingyuan City, Mentougou District, Fangshan District, Lingqiu County, and the southern part of Taihang Mountain. In both degraded and improved areas, factors beyond temperature and precipitation play a significant role. Therefore, it is insufficient to rely solely on residual analysis. It is necessary to quantify additional environmental and human activity factors.

#### Analysis of the evolution of FVC based on OPGD using multiple driving mechanisms

Table 3 shows the eighteen evaluation indexes selected from five categories: primary net productivity of vegetation, meteorological factors, topographic factors, human activity, and social economy. The study employed five classification methods: equidistance classification, natural discontinuous point classification, quantile distance classification, geometric distance classification, and standard deviation distance classification. The classification methods used discontinuous intervals ranging from 4 to 9 categories. Calculate q-values at various discontinuity levels and select the parameter combination with the highest q-value.

**Table 3:** Factors influencing changes in regional vegetation FVC.

Code	Explanatory variable	Code	Explanatory variable	Code	Explanatory variable
X_1_	NPP	X_7_	Proportion of GDP in the tertiary sector	X_13_	Aspect
X_2_	Population	X_8_	Annual cumulative precipitation	X_14_	Type of land use
X_3_	GDP	X_9_	Average annual temperature	X_15_	Soil type
X_4_	Percentage of urban population	X_10_	Night light data	X_16_	Vegetation type
X_5_	Proportion of GDP in the primary sector	X_11_	Elevation	X_17_	Evapotranspiration
X_6_	Proportion of GDP in the secondary sector	X_12_	Gradient	X_18_	Surface temperature

**Figure 7:**
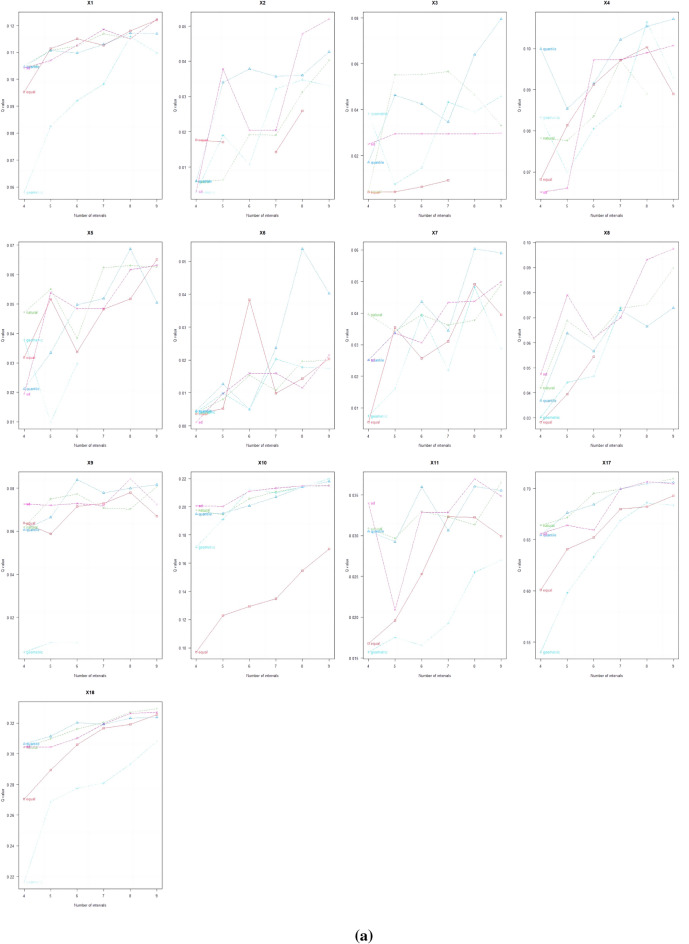

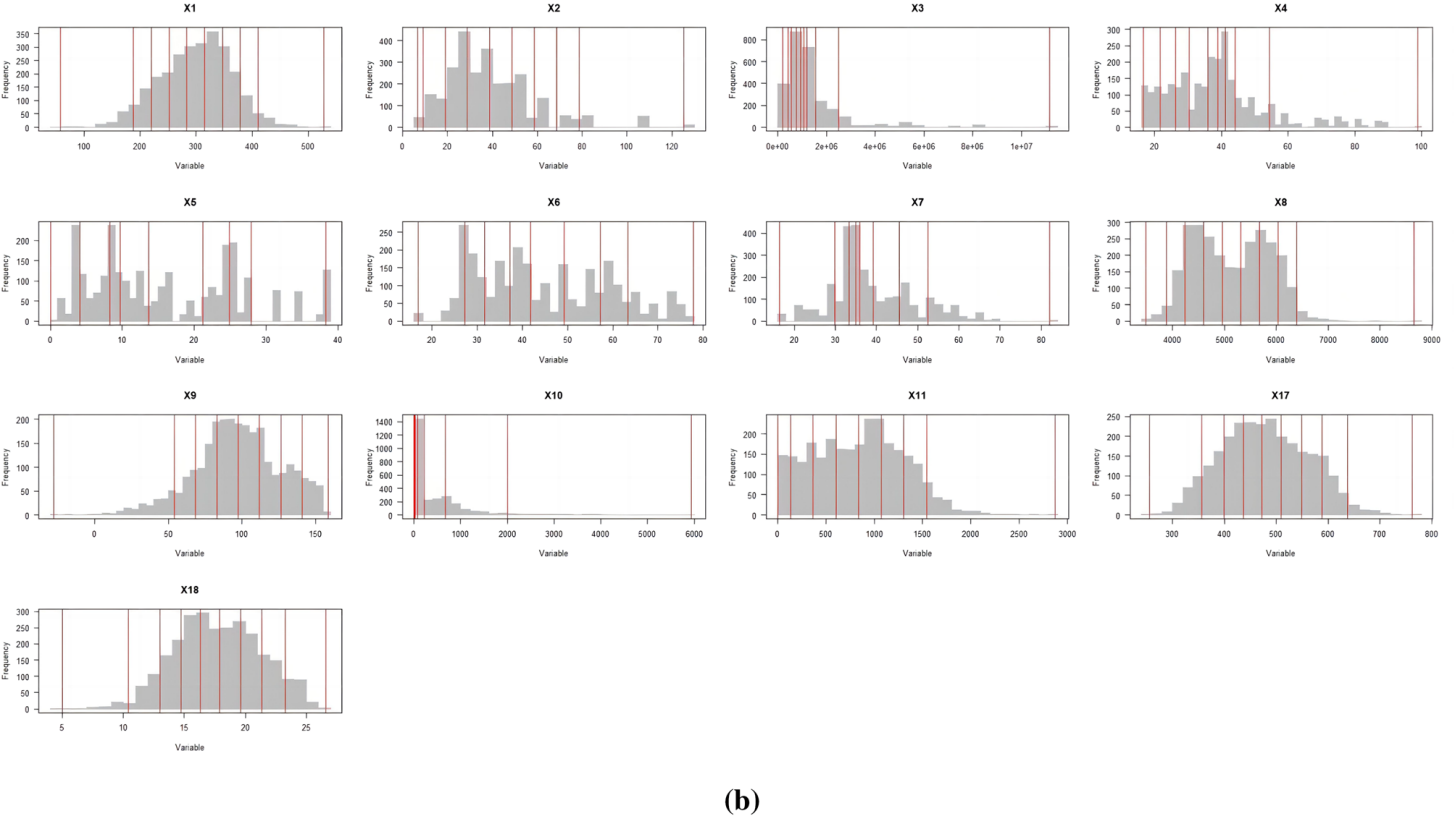
Parameter optimization process a and result b for spatial data discretization.

Figure 7 shows that the discretization process of explanatory variables uses different methods and break points. For NPP (X1), population (X2), and annual cumulative precipitation (X8), it is recommended to choose the standard deviation distance and 9 categories of classification digits. For GDP (X3) and urban population ratio (X4), it is suggested to choose the quantile spacing with 9 categories. For selecting quantile spacing, it is recommended to use X5, X6, and X7, which represent the proportion of GDP in the first, second, and third products respectively. The number of categories should be 8. For selecting standard deviation spacing, it is recommended to use X9, which represents the annual mean temperature, and X11, which represents the elevation. Again, the number of categories should be 8. For X10 (night light data), it is recommended to use geometric spacing with 9 categories for classification. For X17 (evapotranspiration) and X18 (surface temperature), it is recommended to use natural spacing with 9 classifications.

**Figure 8:**
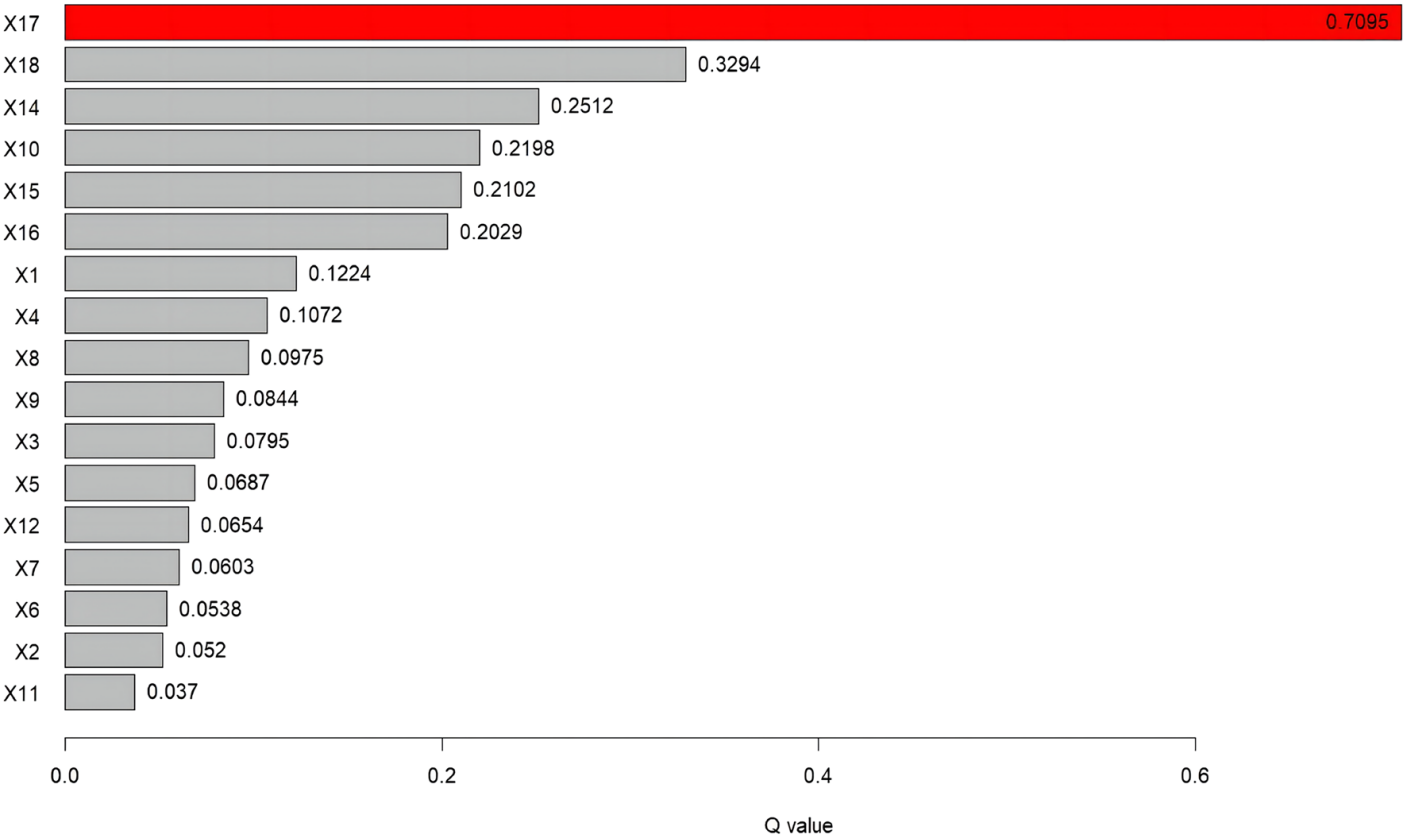
Contribution of single-factor variables to FVC change (p < 0.05).

Figure 8 shows the contribution rate (q value) of different driving factors to FVC in Taihang-Yanshan Mountains from 2000 to 2021. The q value of X17 (evapotranspiration) is 0.71, indicating that it is the driving factor with the strongest explanatory power for FVC spatial differentiation. This suggests that it is the main natural factor affecting the change of FVC. The variables X14 (land use type), X15 (soil type), X16 (vegetation type), X18 (surface temperature) and X10 (night light data) were found to have an explanatory power of over 20%, indicating their significant contribution to changes in FVC. The explanatory power of NPP (X1) and urban population ratio (X4) exceeds 10%. The q value of elevation (X11) is the lowest at 0.037, with an explanatory power of less than 5%. Therefore, the main natural and human factors affecting the change of FVC are considered to be evapotranspiration, land use type, soil type, vegetation type, surface temperature, and night light data.

**Figure 9:**
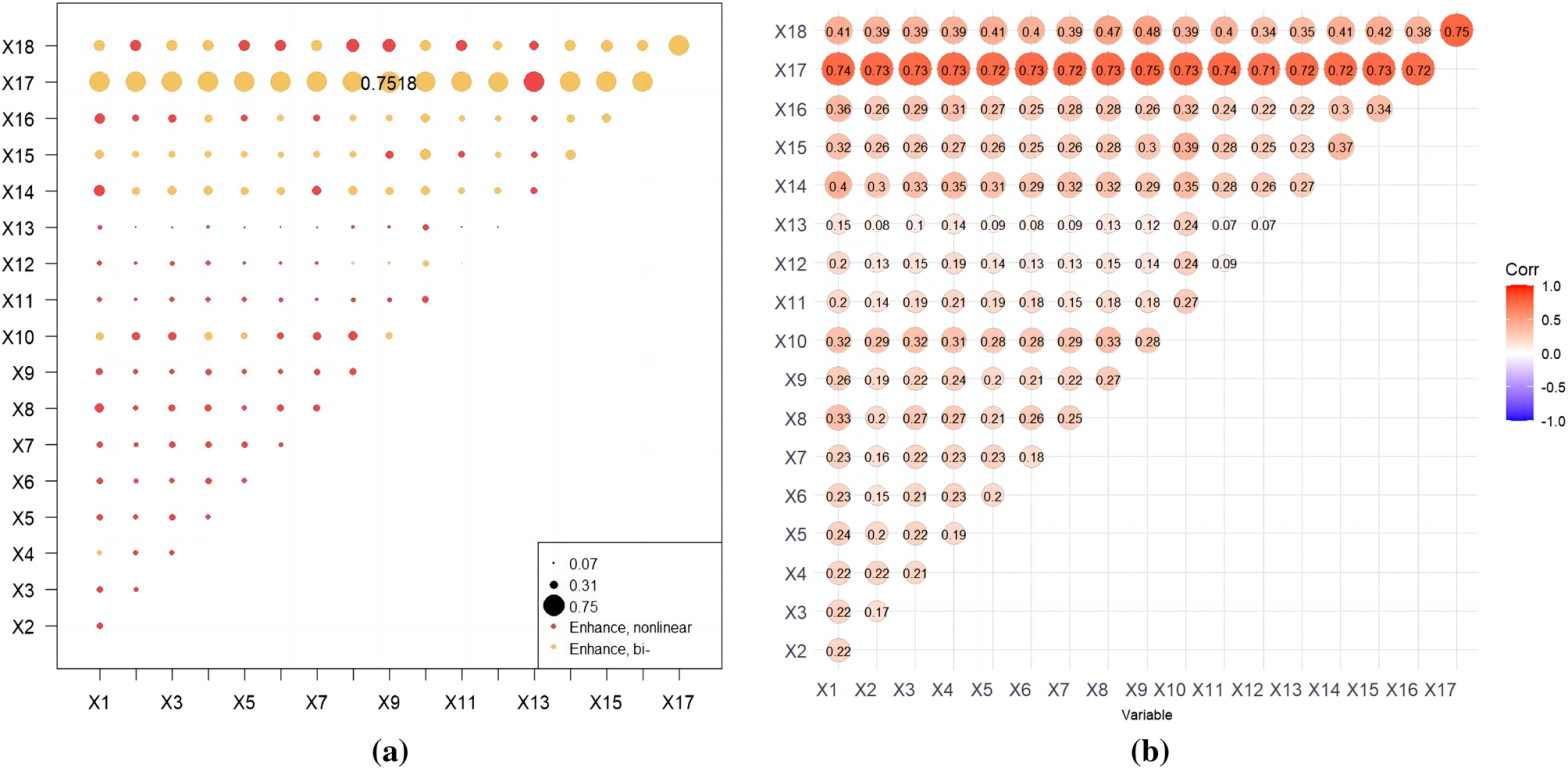
The contribution degree and action form of two-factor interaction.

When analysing the two-factor interaction effect, it was found that the combination of factors that affect the change of FVC is non-linear enhancement and two-factor enhancement, and there is no independent or weakened relationship. The results in Fig. 9 show that the maximum value is X17 ∩ X9 = 0.7518, indicating dual-factor enhancement. The minimum values are X13 ∩ X11 and X13 ∩ X12, both of which have q values of 0.07, indicating non-linear enhancement. Furthermore, when examining the interaction between evapotranspiration, land use, soil type, vegetation, surface temperature, and night light data, it is evident that while both the two-factor and nonlinear enhancement effects are significant, the former holds greater importance. For instance, the interaction between X17 and X9 (0.7518) is stronger than that of X17 and X14 (0.72), X18 and X14 (0.41), and X15 and X10 (0.39), indicating the direction and strength of their combined influence on FVC change. Likewise, interactions involving other influencing factors exhibited distinct patterns of enhancement effects. For instance, the impact of X17 ∩ X1 (0.74) differs from that of X17 ∩ X5 (0.72), X17 ∩ X12 (0.71), and so on. These slight variations in the direction and strength of the interactions highlight the complexity of the relationship that affects the change in FVC. It is important to note that the influence of a pair of factors on FVC changes is not independent, and the combined effect of any two factors surpasses the individual effect of a single factor. Furthermore, the alteration of FVC is significantly influenced by the interaction between natural and human factors. Although elevation alone has explanatory power of less than 5%, its combined effect with other variables exhibits a significant nonlinear enhancement effect, indicating their substantial influence on the change of FVC.

**Figure 10:**
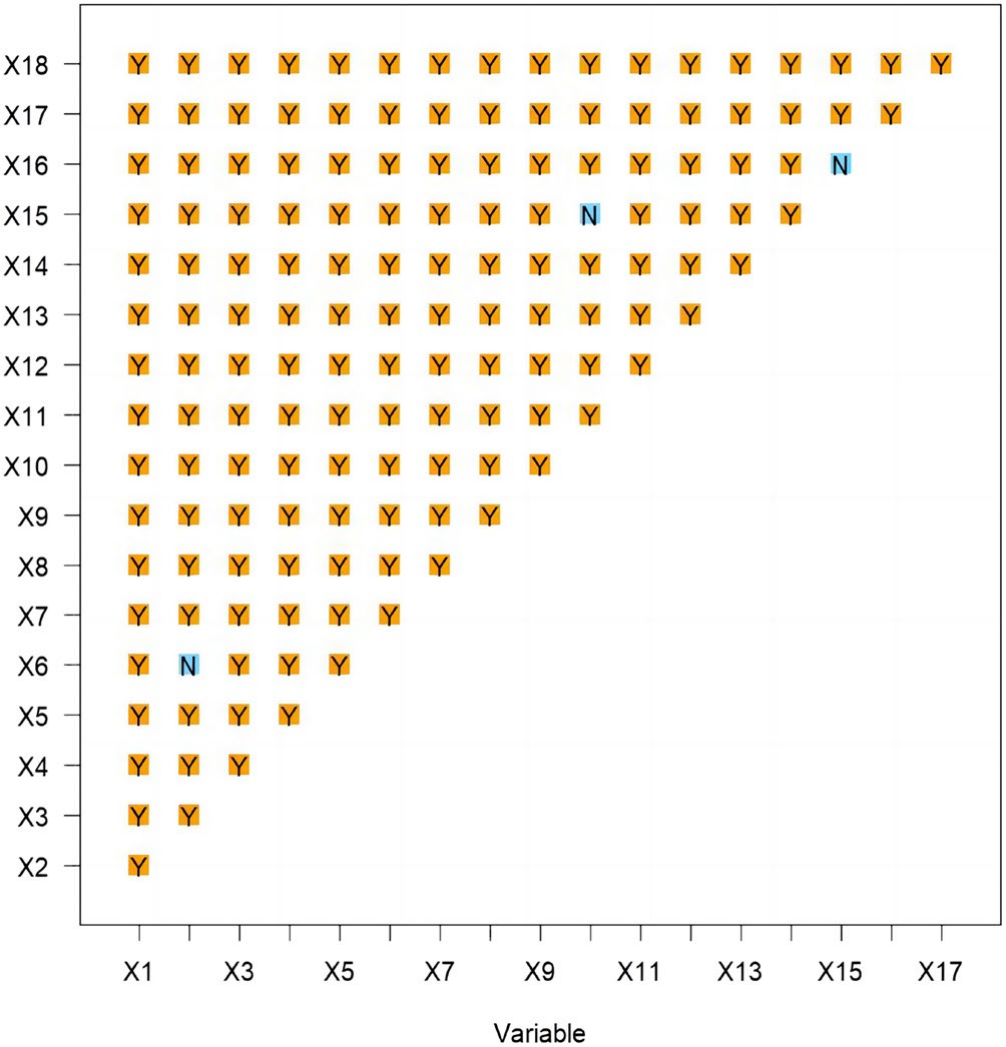
Ecological detection results.

Figure 10 allows for the assessment of significant differences in the degree of influence of each explanatory variable on FVC. Differences between the row and column factors are represented as 'Y' if significant and 'N' if not. Apart from population and the proportion of GDP in the secondary industry, as well as night light data and the three driving factors of soil type, soil type, and vegetation type, the other two factors exhibit significant differences in the spatio-temporal evolution of FVC. The emergence of bilinear and nonlinear enhancement effects can be attributed to the high complexity and rich diversity of ecosystems. Ecosystems are complex networks of interactions between living and non-living components. These interactions are influenced by various ecological mechanisms, biodiversity, and environmental gradients, resulting in diverse forms of enhancement.

**Figure 11:**
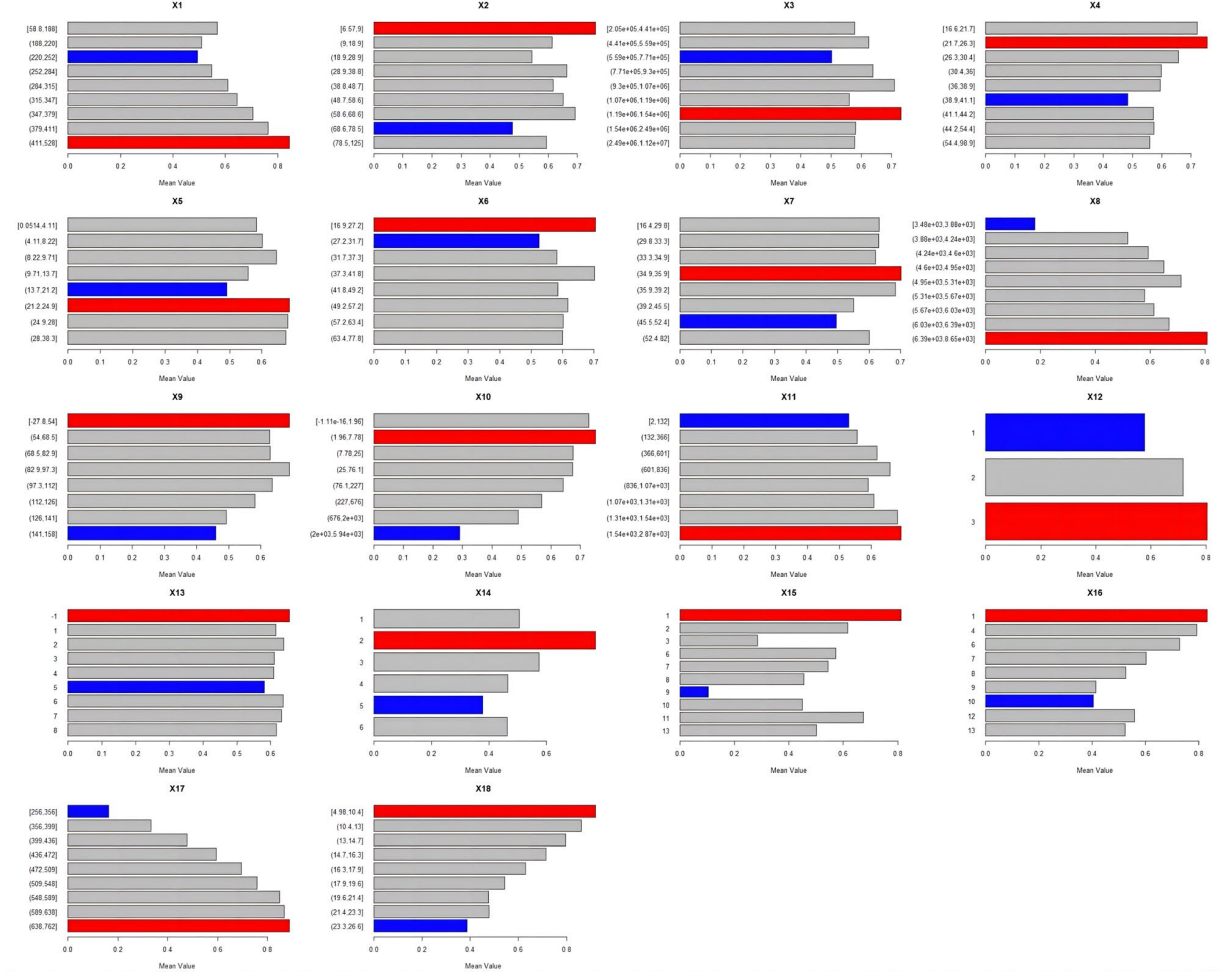
Results of risk detection.

The risk detection results (Fig. 11) use a colour scheme to represent the relative values: red for high, blue for low, and gray for middle. The FVC values for each interval of explanatory variables showed clear differences, with FVC values increasing as slope, elevation, and evapotranspiration increased. The slope has an inclination of 15°–25°, while the altitude ranges from 1540 to 2870 m. The evapotranspiration rate is between 638and 762 mm, and the FVC is above 0.7, indicating high vegetation coverage. The relationship between vegetation growth and the natural environment, topography, and agricultural culture of Taihang-Yanshan Mountain is evident in the steeper terrain at higher altitudes. However, lower elevations tend to have flatter terrain. Hills and valleys are often concentrated areas of human activity, with urban construction and agricultural production causing greater interference and damage to the ecological environment, resulting in lower vegetation coverage. Precipitation and temperature are the primary meteorological factors affecting FVC. The annual cumulative precipitation has a positive correlation with FVC, while the annual mean air temperature and surface temperature have a negative correlation. The FVC value is the largest when the annual cumulative precipitation is between 6390 and 8650 mm, the annual mean air temperature is between − 2.7 and 5.4 °C, and the surface temperature is between 4.98 and 10.4 °C. The data indicates that an increase in precipitation and a decrease in temperature have a positive impact on the growth of FVC. The land use type with the highest FVC was forest land, the soil type with the highest FVC was alluvial soil, and the vegetation type with the highest FVC was evergreen coniferous forest.

#### Correlation analysis was conducted to examine the relationship between county FVC changes and influencing factors using MGWR

**Figure 12:**
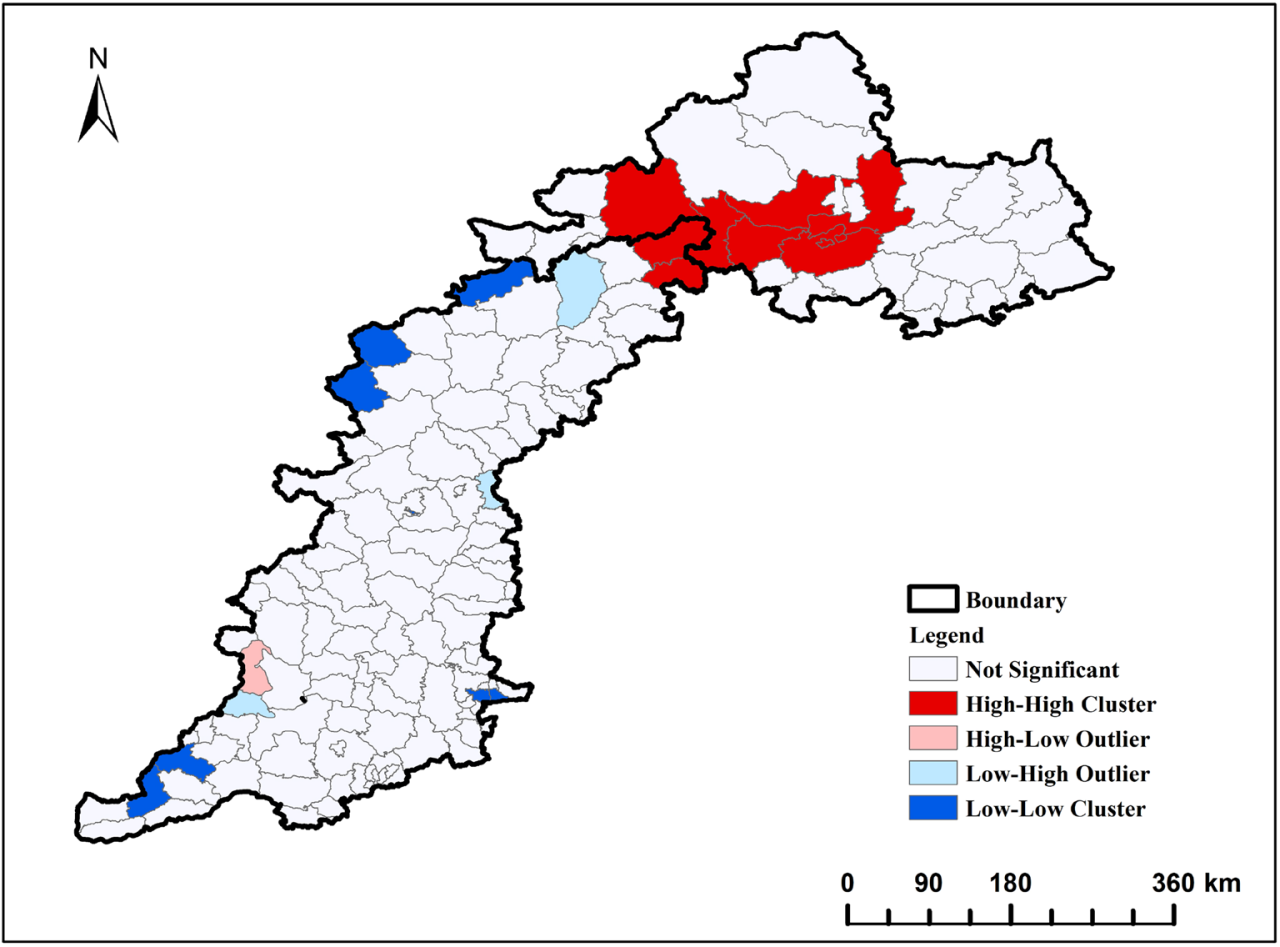
FVC local Moreland index.

The global Moreland index was used to measure the spatial autocorrelation of FVC in the TYR. The P-value was less than 0.01 and the Z-score was 9.006 (greater than 2.58), reaching the 99% confidence interval, and the Moreland index was 0.505 and less than 0.7 (Table 4), indicating that the spatial autocorrelation of FVC existed among counties. The local Molan index can further reveal the degree of aggregation and dispersion of local areas. As shown in Fig. 12, Chicheng County, Changping District, Yanqing District, Luanping County and Xinglong County in the northern part of the study area showed a "high-high" aggregation pattern, indicating good vegetation growth and high FVC. The "high-low" dispersion pattern was concentrated in Guxian District of Linfen City, indicating that the FVC in this area was higher than in the surrounding area. Zhuolu County, Luquan District and Fushan County showed a "low–high" dispersion pattern, indicating good vegetation cover around them, while the "low-low" aggregation pattern was concentrated in Yangquan County, Daixian County, Yuncheng Salt Lake District, Wencheng County and Anyang County, Henan Province, indicating poor vegetation growth.

**Table 4:** FVC Global Moran index.

Variable	Moran’ I	Z-score	P value
FVC	0.505	9.006	0.000

Fourteen continuous variables were selected, including NPP, climate (annual cumulative precipitation, annual average temperature, evapotranspiration, and surface temperature), terrain (elevation and slope), and society (population, GDP, urban population proportion, GDP proportion of primary, secondary, and tertiary industries, and night lighting data). The language used is clear, objective, and value-neutral, and the technical terms are consistent throughout the text. The sentence structure is simple and logical, and the text is free from grammatical errors, spelling mistakes, and punctuation errors. No changes in content were made as per the instructions. The VIF (variance inflation factor) is used to check for collinearity. A VIF value greater than 10 indicates the presence of multicollinearity between this variable and other variables, resulting in information redundancy during the research process. Therefore, six variables were removed from the model: surface temperature, evapotranspiration, annual cumulative precipitation, the proportion of tertiary industry GDP, NPP, and annual average temperature (Table 5). The remaining eight driving factors were used to construct a multi-scale geographical weighted regression model. The regression coefficients were divided into six grades to analyze their spatial heterogeneity.

**Table 5:** Collinearity check.

Variable	VIF	Variable	VIF	Variable	VIF	Variable	VIF
X14	551.207502	X1	32.021682	X4	8.488830	X11	7.835354
X13	148.79188	X9	14.150904	X5	4.203772	X12	7.465248
X8	97.843073	X2	2.897657	X6	4.166219		
X7	56.521051	X3	4.869703	X10	2.080901		

**Figure 13:**
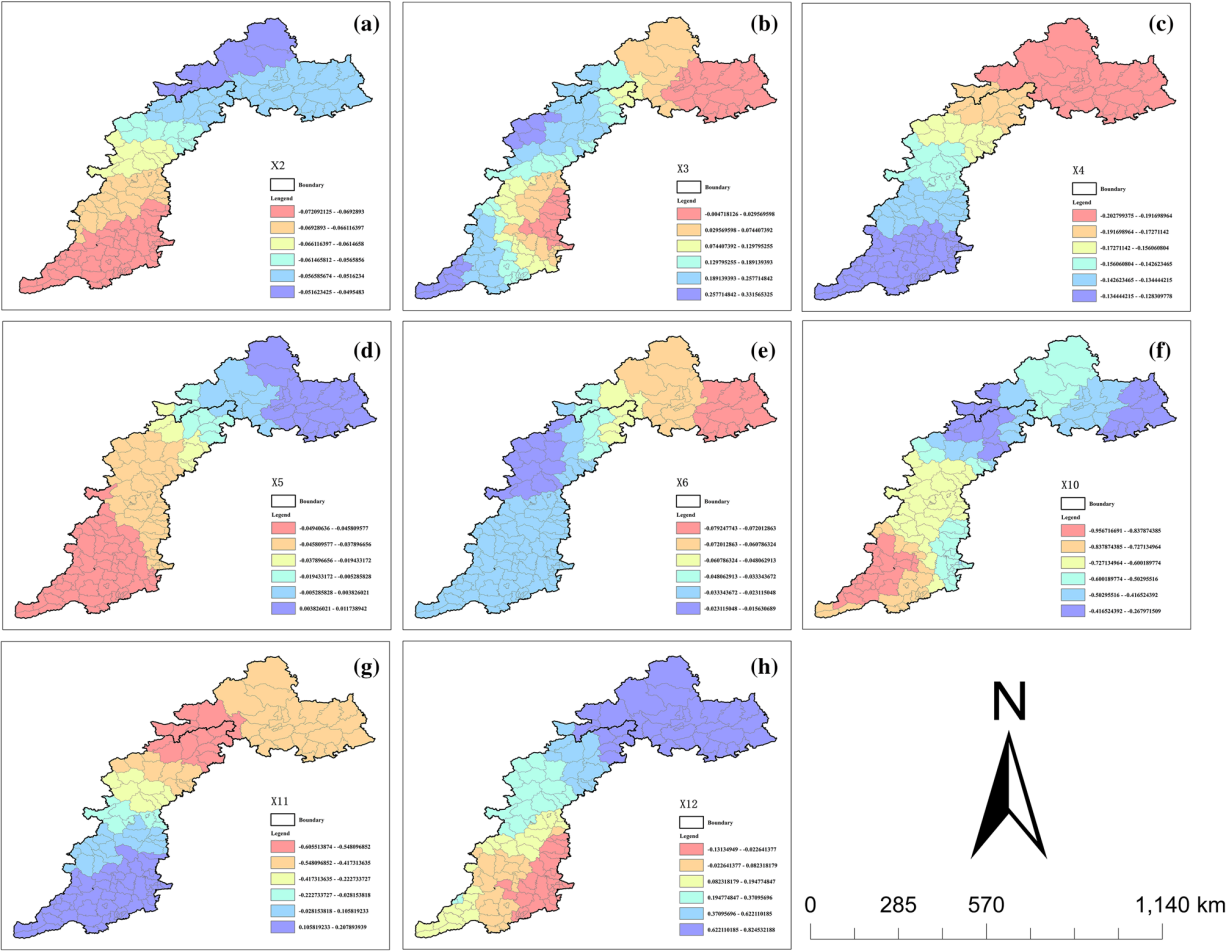
Correlation between FVC changes and variables in each county.

Figure 13 shows that population (X2) has a negative correlation with vegetation response in the study area. This is demonstrated by the gradual increase in spatial distribution from south to north. It is important to note that this analysis is based solely on objective data and does not include any subjective evaluations. However, population has a small overall impact on FVC. The impact of GDP (X3) on FVC is relatively complex, with the regression coefficient ranging from − 0.004 to 0.331. It shows a weak promoting effect in most regions, and only a weak inhibiting effect in Qinglong Manchu Autonomous County, Pingquan County, Xinglong County, Qian'an City, and Luanzhou City in the northeast of the study area, as well as Zanhuang County, Shshe County, and Anyang County in the southeast of the study area. The negative impact of the proportion of urban population (X4) on FVC is greater than that of the number of population. This is particularly evident in the counties located in the northeast of the study area, where the negative correlation coefficient reaches − 0.202. The regression coefficient for the ratio of GDP of the primary industry (X5) and FVC showed a mixed effect, both positive and negative, with a range of variation from − 0.049 to 0.003. This indicates a spatial distribution pattern with higher values in the northeast and lower values in the southwest. The negative effect of X6 on the GDP of the secondary industry is greater than that of X5, particularly in Funing District, Qianxi County, Qian'an County, Pingyuan County, Jianchang County, and Suizhong County. The distribution pattern of night light data (X10) on FVC is clearly heterogeneous, with low values in the southwest and high values in the northeast. This pattern is closely related to population density, urbanization, and the development of primary, secondary, and tertiary industries.

Regarding the topological factors, the impact of elevation (X11) on FVC is weakly positive and strongly negative overall. The regression coefficient varies from − 0.605 to 0.105, indicating a spatial distribution pattern of high in the southeast and low in the northwest. The area of high value is primarily located in the southern part of the study area, while the low value area is concentrated in Chicheng County, Chongli District, Changping District, Fangshan District, and Laishui County. The regression coefficient for the spatial heterogeneity of slope (X12) ranged from − 0.131 to − 0.824 and was found to be significant. The low-value area was located in Lincheng County, Neiqiu County, Wuan City, Cixian County, and Zhangzi County, while the high-value area was distributed in the northeastern part of the study area. This distribution pattern shows a high north and low south spatial distribution. The central and southern plain area has a low slope, making it suitable for human habitation and frequent human activities. However, excessive human intervention can limit vegetation growth and further affect vegetation coverage, leading to a risk of FVC reduction.

The FVC exhibited a significant difference in response to each explanatory variable, with the night light data, elevation, and slope being the most sensitive factors. The results showed distinct spatial heterogeneity. The proportion of secondary industry GDP, GDP, elevation, and slope exhibited positive and negative interactions. The population size, urban population proportion, GDP proportion in primary and secondary industries, and night light data all have negative effects. The night light data has a particularly significant impact.

## Discussion

### Spatial and temporal evolutionary patterns of FVC

The annual mean FVC values between 2000 and 2021 ranged from 0.540 to 0.630, with a steady fluctuation. The sharp decline in FVC in 2009 may be related to the effects of the winter of 2008 and the drought of 2009^39^, which resulted in decreased soil moisture due to dry weather, thus affecting plant growth and development. Before 2012, FVC showed an increasing trend with a linear rate of increase of 0.0022 per year; several ecological protection projects were implemented in the Beijing-Tianjin-Hebei region during this period to improve vegetation conditions. These projects include the Sanbei Protective Forest Project in 1978, the Taihang Mountain Greening Project in 1986, the Beijing-Tianjin Wind and Sand Source Management Project initiated in 2002, and the National Coastal Protection Forest System Construction Project from 2006 to 2015. The growth and coverage of natural vegetation has gradually improved through the implementation of afforestation-based ecological construction projects. However, after 2012, FVC showed a decreasing trend with a linear rate of decline of 0. 0024/a; It may be due to the huge damage caused to the Beijing-Tianjin-Hebei region after the extraordinarily heavy rainstorms in July 2012 hit northern China;At the same time, the 18th Party Congress in 2012 proposed to build a moderately prosperous society in all respects, and vigorously promoted economic construction and social development, and then, as the level of development continued to rise, some areas failed to coordinate the relationship between the economy and the ecology, resulting in the destruction of the ecological environment and a decline in the level of vegetation cover;The vegetation cover in the study area shows the distribution characteristics of high in the north and south and low in the middle, the higher areas are Chengde and Baoding cities, which may be related to the long-term construction of local ecological environment protection as well as the development of green agriculture, and the lower areas are close to the Loess Plateau and Inner Mongolia Plateau, which have a poorer ecological environment. The southernmost part of the Rowan Mountains has higher FVC values on average throughout the year, which may be related to the fact that the vegetation is dominated by evergreen forests such as greasewood, sidecar, and whitebark pine, and that the growth characteristics of the vegetation are related to a number of factors such as its geographic location, climatic conditions, and topographic and geomorphological features. The areas with significant reductions in vegetation cover are mainly located in the eastern Taihang Mountains and the south-central Yanshan Mountains, which may be due to the fragile ecological environment in these areas, and with the regional economic development and accelerated urbanisation, the expansion of land for construction encroaches on forested land, grassland, and arable land, which has an inhibitory effect on the recovery of vegetation^40^. The trend of vegetation cover is closely related to the effects of soil type, vegetation type, elevation, and anthropogenic factors^41^.In the case of significantly reduced areas, not only natural factors but also the impact of human factors should be fully taken into account when protecting and restoring vegetation cover. The greater the number of ecological projects, the greater the proportion of area with improved vegetation cover.

### Analysis of the relative contribution of climate change and other factors to the evolution of FVC

Studies on vegetation changes and their influencing factors, climate change and other factors are often considered to be the most dominant influencing factors^42^. Residual analysis was used to separate the different contributions of climatic factors from other factors to vegetation cover. It was found that the degraded areas were concentrated in the southern Yanshan Mountains and the eastern Taihang Mountains, while the other areas were scattered, which may be related to climate, topography, anthropogenic activities, and errors in calculations^43^, taking into account a variety of factors. Degraded and ameliorated areas, which are affected by a combination of climate change and other factors, account for 69.75 per cent and 74.25% of the area, respectively, and are the main drivers of changes in FVC. In the degraded and improved areas, climatic factors alone play a small role, while other factors dominate, occupying 76.78% and 80.80% of their areas, respectively, and it is speculated that there are two main reasons for this phenomenon, on the one hand, thanks to the fact that human beings carry out a large number of environmental protection works^44,45^, and on the other hand, the reason for the The residual analysis data used diverse data sources, which may have differences in data standards and collection time and place, further leading to possible errors in the results^46^. Furthermore, in addition to the effects of human activities, there may be other factors, such as extreme climatic, topographic and hydrological conditions, which may cause errors in the results.The results of the residual analysis indicate that factors other than temperature and precipitation are the primary drivers of FVC changes in the TYR, a result that is consistent with previous studies in regions such as the North China Plain^47^ and the Yangtze River Delta^48^. To a certain extent, it shows that in the process of analysing the data, attention should be paid to the precise control of logarithms and experimental methods, as well as the negative impact of other factors on vegetation in urban areas^49^. The above influences are only some of the factors other than precipitation and temperature, and the driving mechanisms for changes in vegetation cover are exceptionally complex, and quantitative studies of other specific factors are urgently needed, hence the subsequent in-depth analyses and discussions using OPGD and MGWR.

### Multiple driving mechanisms for the evolution of FVC

The continuous type variables were discretised by the parameter optimal geoprobe technique to filter out the optimal intermittent methods and categories. The results of factor detection showed that the spatial differentiation of FVC in the TYR was jointly influenced by topographic factors, climatic factors, human activities and land use types, but in general the influence of natural factors on FVC was stronger than that of human factors. The q-value of evapotranspiration was the largest, reaching 0.7095, indicating that it was the dominant factor affecting FVC, and the increase of evapotranspiration would enhance the microbial activity in the soil layer, thus accelerating the rate of decomposition of soil organic matter, which was favourable to the growth of the vegetation root system^50^. In addition, factors such as surface temperature, land use type, and nighttime lighting data had greater than 20 per cent explanatory power; Surface temperature can directly affect the metabolic processes of photosynthesis, respiration and transpiration of vegetation, land use type can effectively reflect the regional differences in land use patterns, vegetation types, and the degree of human activities, and the higher intensity of nighttime lighting represents the higher level of regional economic development to a certain extent, so the explanatory power of these factors for spatial differentiation in FVC in the TYR is also higher, which is in line with the results of existing studies consistent^51,52^; Interaction probes showed that the determining power after the interaction of two factors was greater than that of a single factor, and both factors showed nonlinear enhancement or two-factor enhancement, further proving that the spatial distribution pattern of FVC in the TYR was affected by a variety of factors, and that the explanatory power generated by the interactions of evapotranspiration and other factors was above 0.7, of which that with the average annual temperature had the strongest explanatory power for FVC, with a q value of 0.7518. Surface temperature also interacted strongly with evapotranspiration, with a q-value of 0.75, which suggests that temperature has a role to play in the influence of FVC, and evapotranspiration interacted with land-use type and nighttime lighting data to a lesser extent, with q-values of 0.72 and 0.73, which reflects the fact that human activities are an important factor influencing the FVC of the TYR^53^; On the one hand, ecological restoration projects such as returning farmland to forests and grasslands can contribute to the recovery of vegetation, while on the other hand, accelerated urbanisation and excessive resource exploitation can degrade the vegetation^54^; It is worth noting that the explanatory power of elevation for FVC in the TYR is only 0.037, but its explanatory power after interaction with evapotranspiration can be as high as 0.74, indicating that elevation can also have a significant effect on FVC after satisfying certain evapotranspiration conditions. The results of the ecological and risk probes show that, with the exception of three groups of factors—population size and secondary GDP share, nighttime light data and soil type, and soil type and vegetation type—the other factors show significant differences in the spatial and temporal evolution of FVC. For climatic factors, risk detection in terms of annual precipitation shows that vegetation cover increases as precipitation increases, with the highest vegetation cover in Subregion 9 when precipitation reaches 6390–8650 mm. This also reflects the severity of drought conditions in the TYR, as plants require sufficient water for growth^55^. Vegetation cover showed a tendency to increase and then decrease as the mean annual temperature increased, especially in the first subregion, which had the highest vegetation cover when the mean annual temperature ranged from − 27.8 to 54 °C. The results of the risk detection of surface temperatures show that an increase in surface temperatures does not promote the growth of vegetation in the area because an increase in surface temperatures leads to a decrease in the amount of water in the soil, which inhibits the growth of plants. In contrast, good coupling of hydrothermal conditions contributes to vegetation growth, especially in Division 9, where evapotranspiration reaches 638–762 mm, with maximum FVC; In terms of topographic factors, the range of slopes suitable for vegetation growth is between 15° and 25°, and the vegetation cover appears to show a trend of first increasing, then decreasing and then increasing with the increase of elevation, it is obvious that the magnitude of slopes has a greater influence on the change of the vegetation cover in the TYR, whereas the influence of slope direction on the growth status of the vegetation is relatively small, and the lowest FVC is found in the flat land, whereas the difference of the FVC in the other topographic subregions is were less obvious; In terms of soil factors, different soil types and vegetation types also affect the distribution of vegetation cover in the region. For example, vegetation cover is highest when the soil type is leachate and the vegetation type is evergreen coniferous forest, and lowest when the soil type is saline and the vegetation type is vegetation on urban and built-up land. These results results suggest that while focusing on the effect of a single factor on FVC, it is important to pay more attention to the positive and negative effects of interactions between factors on FVC. There are differences in the suitable characteristics of each influencing factor for FVC in different geomorphological units. The suitable range or type of factors is crucial for vegetation growth, which can improve the science and accuracy of quantitative analysis and identification of vegetation drivers, as well as being a key factor influencing the spatial distribution of vegetation cover. Therefore, when formulating the ecological conservation plan, it is necessary to consider the appropriate range of FVC for each impact factor in different geomorphological units in order to achieve site-specific ecological conservation. This is of great significance in enhancing the effectiveness of ecological civilisation construction in the TYR and promoting the harmonious coexistence of man and nature.

It has been shown that human activities and natural environment jointly affect FVC, but there is a lack of exploration of the causes of spatial heterogeneity of FVC under the joint effect of human activities and natural environment, without in-depth consideration of the differences in the changes of multiple variables at the spatial scale, while the MGWR model can reflect the differences in the scales of the regression parameters of the multivariate variables, and improve the accuracy of the response mechanism of the different variables, and improve the accuracy^56^. Therefore, the MGWR model was used to analyse the law of spatial heterogeneity of FVC at the county scale in the TYR, which to a certain extent bridges the gap of multivariate-driven research on vegetation cover in the TYR, and spatial autocorrelation analyses were carried out by using the global Moran's index and the local Moran's index, to ensure that the selected variables are correlated to FVC and to explore the degree of their correlation; The redundancy analysis was also combined with VIF to test whether there are multiple covariance characteristics among the variables, among which six variables, namely, surface temperature, evapotranspiration, annual cumulative precipitation, annual mean temperature, NPP, and GDP share of the tertiary industry, have stronger covariance, and the screened variables were used with the MGWR model to enhance the explanatory power of the FVC, and to illustrate the spatial differentiation characteristics of them with the FVC. The results show that the response of FVC to different variables in the study area is very different, in which GDP, the proportion of GDP in the secondary industry, elevation and slope show positive and negative effects interactively, but in most of the area show negative inhibitory effects, GDP can reflect the state of economic development of a region, and the greater the intensity of human activities, the stronger the destruction of the vegetation cover of the ground surface^57^; The topographic factors show a spatial pattern of high in the north-east and low in the south-west, and in the south-central plains area there is little restriction by the topography, the terrain is gentle and suitable for human habitation, and there are frequent human activities, facing the possibility of a decrease in FVC, and the northeastern hilly area is actively carrying out ecological engineering construction projects, which play a certain role in the promotion of FVC^58^; Population size, the proportion of urban population, the proportion of GDP in the primary and secondary industries, and night-time lighting data are all negatively inhibited, and the night-time lighting data are particularly significant, the spatial heterogeneity of the FVC is obvious, manifesting itself in a distribution pattern of low in the southwest and high in the northeast, night-time lighting is mostly from the city-wide man-made sources, and the differences in human night-time activities can, to a certain extent, reflect the inter-regional development situation. In the process of rapid urbanisation, human activities have led to a decline in vegetation cover, revealing in depth the strong driving force of human activities on vegetation degradation^59^, suggesting that human activities have a strong driving force on vegetation, attaching importance to its dominant role in vegetation restoration, strengthening the construction of an ecological civilisation, and actively implementing ecosystem protection and restoration projects to comprehensively promote the construction of a beautiful China.A total of 18 explanatory factors were selected for study in this paper, but there are still some factors (e.g., groundwater storage, solar radiation, wind speed, wind direction, etc.) that were not included, which makes the results incomplete.

## Conclusions

This paper examines the spatial and temporal variation characteristics of FVC in the TYR from 2000 to 2021. It employs Sen trend analysis to investigate the multivariate driving mechanism of FVC evolution in the study area. This is achieved through residual analysis, parametric optimal geoprobe, and multi-scale geographically weighted regression. The main conclusions are as follows:

1. The FVC in the TYR demonstrates a gradual and fluctuating upward trend, with an average growth rate of 0.02/10a. The spatial pattern of this trend is characterised by a high concentration in the northwest and a low concentration in the southeast. Between the years 2000 and 2021, more than half of the study area exhibited an increase in FVC on an annual basis. This increase was particularly pronounced in the western part of the Yanshan Mountains and the central and western parts of the Taihangshan Mountains. The study revealed that more than half of the areas exhibited an annual increase in FVC between 2000 and 2021. This was particularly evident in the western Yanshan Mountains and the central and western Taihang Mountains, where the increase was highly significant. Conversely, 12.24% of the areas exhibited a decreasing trend, with the southern Yanshan Mountains and the eastern Taihang Mountains experiencing the most pronounced decline.
2. In order to ascertain the relative contribution of climate change and other factors to the evolution of FVC, the study area was divided into improved and degraded areas of FVC. It was found that 74.25 per cent and 69.75 per cent of the area, respectively, were jointly influenced by climate change and other factors. Other factors were the dominant influence on 80.80 per cent of the area in the improved zone and up to 76.78 per cent of the degraded zone. It was demonstrated that factors other than climate change were the primary driving force behind the evolution of spatial and temporal patterns of FVC in the TYR.
3. The results of the OPGD analysis indicate that the primary factor influencing FVC in the TYR is evapotranspiration (q = 0.7095). Furthermore, the explanatory power of surface temperature, land-use type, nighttime light data, soil type and vegetation type is greater than 20%, and is least influenced by elevation (q = 0.037). The combined effect of two factors was found to be greater than that of a single factor, with both showing nonlinear enhancement and two-factor enhancement. The two-factor combinations of evapotranspiration and mean annual air temperature, and evapotranspiration and surface temperature were found to have the strongest influence on the spatial and temporal evolution of FVC (q = 0.75). Surface temperature ranged from 4.98 to 10.4 °C, evapotranspiration from 638 to 762 mm/a, and nighttime lighting from 1.96 to 7.78 lm/m^2^, which favoured an increase in vegetation cover. Furthermore, vegetation developed on the gonadal soil was more inclined to high cover. The least effect on FVC was observed for slope direction and elevation (q = 0.07), which demonstrated a non-linear enhancement.
4. The MGWR model was employed to investigate the spatial heterogeneity of multivariate drivers in the TYR. The Moran index was utilized to analyze the spatial autocorrelation, and the covariance test was integrated with the variance expansion factor to identify the pertinent variables. It was determined that the FVC was more responsive to the influences of night-time lighting data, elevation, slope, and other factors. Furthermore, the characteristics of the spatial differentiation were found to be significant. Among these, the GDP, the percentage of the GDP in the secondary industry, the elevation, and the slope were found to have a complex effect on the FVC. The GDP, the proportion of GDP in the secondary industry, elevation and slope exert a complex effect on FVC, which is both positive and negative. The remaining variables exert a negative effect.

## Data Availability

The data presented in this study are available on request from the corresponding author.
